# A European Melting Pot of Harbour Porpoise in the French Atlantic Coasts Inferred from Mitochondrial and Nuclear Data

**DOI:** 10.1371/journal.pone.0044425

**Published:** 2012-09-12

**Authors:** Eric Alfonsi, Sami Hassani, François-Gilles Carpentier, Jean-Yves Le Clec’h, Willy Dabin, Olivier Van Canneyt, Michael C. Fontaine, Jean-Luc Jung

**Affiliations:** 1 Laboratoire BioGeMME (Biologie et Génétique des Mammifères Marins dans leur Environnement), Université Européenne de Bretagne & Université de Bretagne Occidentale, Brest, France; 2 Laboratoire d’Etude des Mammifères Marins, Océanopolis, Brest, France; 3 LEMAR, Université Européenne de Bretagne & Université de Bretagne Occidentale, Brest, France; 4 Observatoire PELAGIS, UMS 3462, CNRS-Université de La Rochelle, La Rochelle, France; 5 Laboratoire d’Ecologie, Systématique et Evolution, Université Paris-Sud – CNRS, Orsay, France; 6 Ecoanthropologie et d’Ethnobiologie, UMR 5145, CNRS-MNHN-Université Paris 7 Musée de l’Homme, Paris, France; University of Canterbury, New Zealand

## Abstract

Field surveys have reported a global shift in harbour porpoise distribution in European waters during the last 15 years, including a return to the Atlantic coasts of France. In this study, we analyzed genetic polymorphisms at a fragment of the mitochondrial control region (mtDNA CR) and 7 nuclear microsatellite loci, for 52 animals stranded and by-caught between 2000 and 2010 along the Atlantic coasts of France. The analysis of nuclear and mitochondrial loci provided contrasting results. The mtDNA revealed two genetically distinct groups, one closely related to the Iberian and African harbour porpoises, and the second related to individuals from the more northern waters of Europe. In contrast, nuclear polymorphisms did not display such a distinction. Nuclear markers suggested that harbour porpoises behaved as a randomly mating population along the Atlantic coasts of France. The difference between the two kinds of markers can be explained by differences in their mode of inheritance, the mtDNA being maternally inherited in contrast to nuclear loci that are bi-parentally inherited. Our results provide evidence that a major proportion of the animals we sampled are admixed individuals from the two genetically distinct populations previously identified along the Iberian coasts and in the North East Atlantic. The French Atlantic coasts are clearly the place where these two previously separated populations of harbour porpoises are now admixing. The present shifts in distribution of harbour porpoises along this coast is likely caused by habitat changes that will need to be further studied.

## Introduction

The harbour porpoise (*Phocoena phocoena*), one of the smallest cetaceans, is widely distributed in the cold to temperate coastal waters of the northern hemisphere. The species occurs in three major areas, the North Pacific, the North Atlantic and the Black Sea [1], [2]. In the North Atlantic Ocean, it is the most common cetacean species [3]. The North Atlantic population of harbour porpoise has recently been the subject of several studies, that focused mainly on its spatial and temporal distribution [3]–[8]. From the 1940s onwards, field observations (based on strandings, by-catch and sightings) reported that harbour porpoises, commonly encountered in the southern North Sea and off the coasts of the European mainland from Spain to Denmark, declined abruptly [9], [10]. More recently, the large scale field surveys SCANS I, performed in 1994 [3] and SCANS II performed in 2005 [5], estimated a constant abundance of about 385,000 harbour porpoises in the eastern part of the North Atlantic [5]. However, a comparison of these survey results also highlighted a marked shift in distribution range of the species in the European waters during a 10-year period. More commonly distributed in the northern part of the North Sea in 1994, the surveys conducted in 2005 detected higher abundances of harbour porpoises along the south-east coast of the United Kingdom and in the Celtic Sea. In the eastern part of the North Atlantic, the harbour porpoises clearly experienced a global southward shift going on for some years.

Local studies confirmed this shift in distribution, and the return of harbour porpoises have been clearly documented along Dutch [11], German [6], [12], Southwest Britain [13] and French coasts [8]. However, the reasons for these global movements are not clearly understood. Reijinders [10] argued that a mix of environmental changes and of direct anthropogenic impacts could be involved (*e.g.* variations in the availability of prey, especially herring and mackerel, and by-catch in fishing nets). Indeed, the repartition of harbour porpoises is expected to be strongly tied to variation in the primary and secondary productivity that provides the basis for apex consumers [14]–[16]. Harbour porpoises display an energy demanding reproductive schedule [14], as females are often gestating and lactating at the same time and parturition occurs shortly before mating [17]. Their small body size also limits their ability to store energy [18]. Taken together, these factors suggest that harbour porpoises must feed frequently without prolonged periods of fasting. Relatively continuous accessibility to adequate prey is therefore critical, and any changes in prey availability may affect energy stores, and ultimately survival [19]. Thus, temporary shortages in prey availability can have negative impacts on these animals and are likely to be responsible for changes in their distribution [20]–[23].

Harbour porpoises also suffer considerable mortality due to accidental by-catches in certain commercial fisheries. For instance, a clear increase in the proportion of by-catch among the stranded harbour porpoises along the coasts of Brittany in North West of France has been observed in the winter months [8]. Although the population of harbour porpoise in the North Atlantic is relatively large, area-specific studies have demonstrated that the impact of by-catch may be worrying [24]–[27]. Moreover, the impact of environmental changes on marine mammal species has also become a major, if not the first preoccupation [16], [28]–[30]. The changes in the distribution patterns of harbour porpoises, which occurred on a very short and recent time scale, certainly illustrate this problem. However, any assessment and conservation efforts require a detailed knowledge of the species abundance and population structure.

Consequently, there has been a growing interest in studying the population structure of the harbour porpoise in recent decades [31]–[34], especially in the North Atlantic waters. In the eastern part of the North Atlantic, previous genetic studies have shown that some genetic differentiation existed between local groups of harbour porpoises [35]–[38], but at the eastern North Atlantic scale most of the species’ distribution range behaved as a continuous population with the genetic differentiation between individuals increasing with the geographic distance (*i.e.* Isolation by distance pattern) [15], [39]. However, this continuum is limited to the south of the Bay of Biscay by marked oceanographic changes, with deep warm waters deviating from the harbour porpoise habitat requirements [15], [16]. Harbour porpoises also occur further south along the Iberian coasts. Fontaine *et al.* [15], [16] showed that Iberian porpoises were a population genetically distinct from the one found further north.

Harbour porpoises are increasingly sighted and stranded over the last 11 years along the French Atlantic coast [8], but at present their origins are unclear. They could originate from the north with a southward shift of porpoises from the Irish Seas, Celtic Seas, and the English Channel. Alternatively, harbour porpoises could have originated from the Iberian population and have crossed the unsuitable habitat conditions in the south of the Bay of Biscay, or form a mixture from these two putative source populations.

In this study, we investigated the genetic compositions of harbour porpoises stranded or by-caught over the last ten years along the coasts of France. We analyzed the genetic polymorphism of these individuals at a fragment of the mtDNA control region and at 7 autosomal microsatellite loci. The maternally inherited mtDNA provides a maternal view of the population structure and diversity. Furthermore, this mtDNA marker has been widely used to study harbour porpoises genetic structure in Europe [31], [32], [38]–[41]. This enabled us to place our local study in a global context. On the other side, fast-evolving bi-parentally inherited microsatellite loci provide a complementary perspective to the mtDNA. These type of markers were also shown to be highly informative to discriminate the Iberian harbour porpoises from those further North [15], [16]. The complementarity between mtDNA and microsatellite loci thus provides a suitable approach to investigating the genetic composition of harbour porpoises increasingly found along the Atlantic coasts of France.

## Results

**Figure 1:**
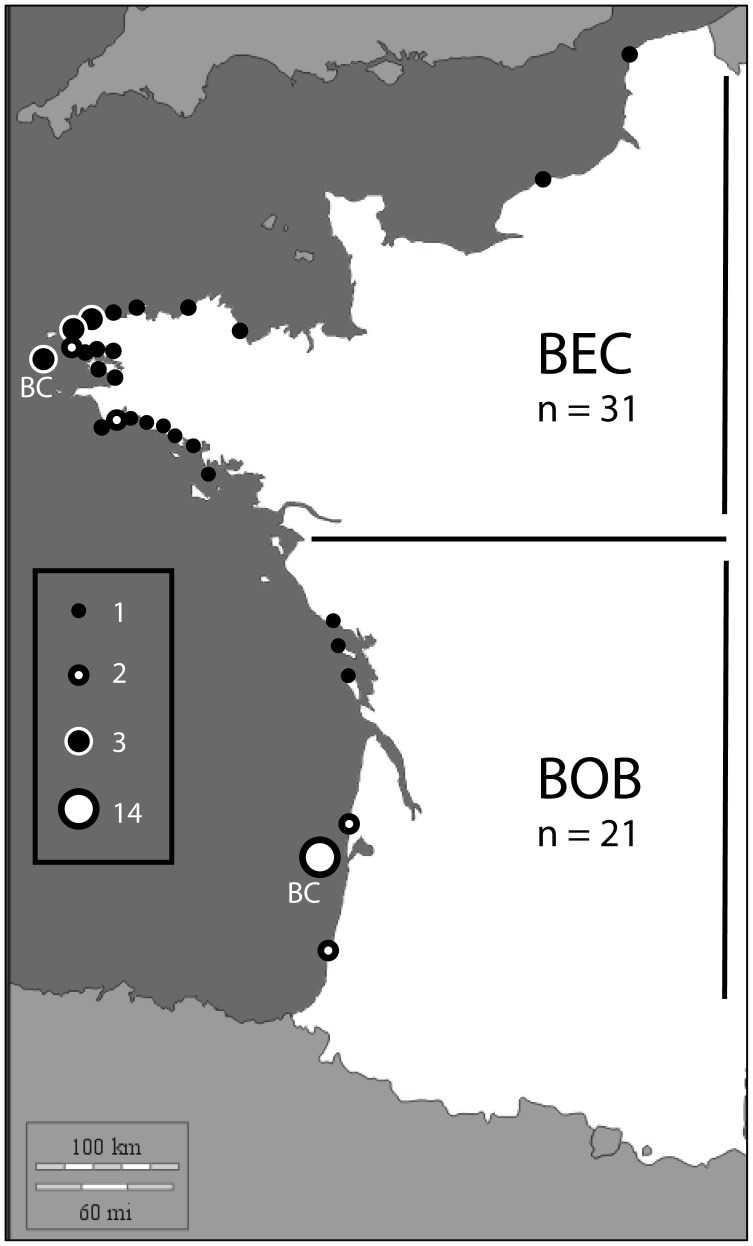
Geographic repartition of sampling sites of harbour porpoises. Samples were taken from 52 stranded or by-caught (BC) harbour porpoises. Dots indicate the places of sampling, and numbers of individuals for each site are indicated. Samples have been arbitrarily named following their place of sampling, either as BOB (Bay Of Biscay) or BEC (Brittany and English Channel).

Tissue samples were collected from 52 harbour porpoises, stranded or by-caught along the French coasts between 2000 and 2010 (Figure 1). None of the samples were included in previous studies (W. Dabin, S. Hassani, personal communication). Two groups of samples were *a priori* defined, depending on the locations of the stranding or catching (Figure 1): one group included the samples of the North of France (group BEC for “Brittany and English Channel”) and the other one those of the south of France (group BOB, “Bay of Biscay”). The two groups were of comparable size, 21 individuals for the BOB group (7 females, 14 males), and 31 individuals for the BEC group (16 females, 15 males) (Table S1).

### Analysis of mtDNA Control Region Sequences

Fifty samples were sequenced for a 581 bp fragment of mtDNA Control Region (mtDNA CR) including also the tRNA-pro and part of the tRNA-thr. Twenty-four variable sites (21 transitions, 1 transversion and 2 deletions) defined 15 unique haplotypes (named from FrA to FrO**,** GenBank Accession Numbers: HQ412579-HQ412587 and JF461056- JF461061; Table S2). Eleven were found in the BEC samples and 8 in the BOB ones. The haplotypes FrM (n = 15), FrL (n = 10), and FrE (n = 10) were the three most common and were found in the two groups of samples (BOB and BEC). Eleven haplotypes were found only once, and the FrG haplotype was identified in 4 individuals of the BEC (n = 3) and the BOB (n = 1) groups. Haplotype (H) and nucleotide (π) diversities overall the sampling were *H_All_* = 0.84 (95Confidence Interval (CI): [0.53–0.91]) and π*_All_ = *0.00638 (95CI:[0.00155–0.0162]) and were comparable in the two geographic groups (*H_BOB_* = 0.85 (95CI:[0.46–0.92]) and H*_BEC_* = 0.84 (95CI:[0.50–0.92]) and π*_BOB_* = 0.00620 (95CI:[0.00121–0.0150]) and π*_BEC_* = 0.00666 (95CI:[0.00167–0.0171]).

**Figure 2:**
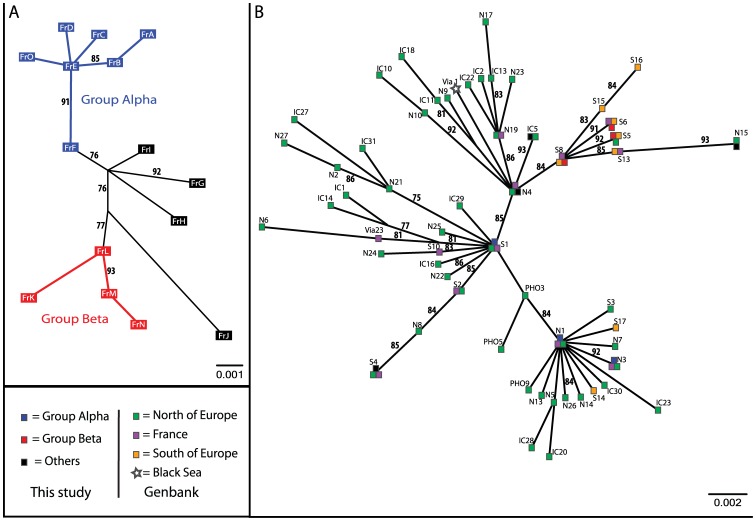
Maximum likelihood trees reconstructed using mtDNA control region haplotypes. A. Maximum likelihood tree obtained for the 581 bp mtDNA control region sequences determined in this study. Supports for the nodes were determined by an approximate likelihood-ratio test, and values are represented in %. Two groups, possessing high branch support values, appeared from the tree analysis (represented as **group** **α** and **group β**), each of the two mixing individuals of the two arbitrary geographic groups BEC and BOB. B. Maximum likelihood tree obtained for the 56 haplotypes defined from the common 334 bp fragment of the harbour porpoise mtDNA control region determined during this study and previous ones (listed on Table S3). Supports for the nodes were determined by an approximate likelihood-ratio test, and values are represented in %. The sequence of the mtDNA control Region of an harbour porpoise coming from black sea (EF063646, [15], [40]), was included in the analysis. Geographic origins of the samples can be seen on the plot. Except 2, all haplotypes found on individuals sampled at positions south of France in previous studies (*i.e.* Iberian and African coasts) are clustered in a group together with the 4 haplotypes of the group β characterized in this study. The group is supported by high value tested by the approximate likelihood-ratio method.

**Figure 3:**
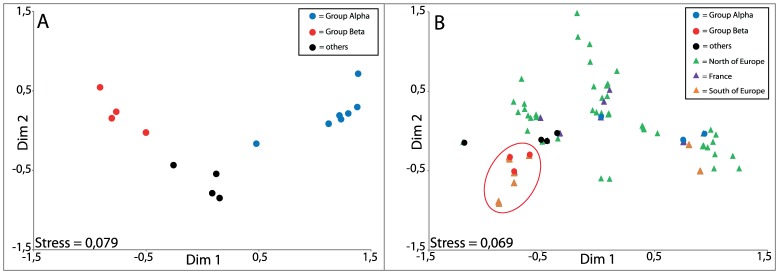
Multidimensional Scaling plots representative of distance between mtDNA control region sequences. **A**. Distances between the harbour porpoise samples analyzed in this study. Individuals attributed to group α by the maximum likelihood analysis are represented as blue dots, to group β as red dots, and individuals unassigned as black dots. Individuals of each group are clearly clustered together, thus highlighting the discrimination between the two groups. **B**. Distances between the 56 haplotypes defined from the common 334 bp sequenced during this study and previous ones (listed on Table S2 and S3). Geographic origins of the samples can be seen on the plot. Except 2, all the haplotypes determined from samples coming from areas localized south to France (Spain, Portugal or African coasts) and from samples of the group β of this study are clustered together.

Phylogenetic relationships between the 15 haplotypes are displayed on an unrooted Maximum Likelihood (ML) tree (Figure 2a) and on a minimum spanning network (Figure S1a). No obvious geographic partitioning of haplotypes was identified. Haplotypes carried by the harbour porpoises from the two geographic groups (BEC and BOB) appeared to be distributed randomly across both the tree and the network. This is consistent with the non-significant value obtained for both measures of genetic differentiation at the haplotypic level (*H_ST_* estimator of Hudson *et al* [42]) and at the nucleotidic level (Hudson’s nearest neighbor distance, *Snn*, [43]) between the two groups tested (BEC and BOB, *H_ST_* = −0.011, p = 0.863; *Snn* = 0.464, p = 0.989). Although no geographic partitioning was observed, two groups of haplotypes can be identified in the haplotype network and in the ML tree. The first group (hereafter called α) displayed a “star-like” topology composed of 6 haplotypes surrounding a dominant haplotype (in blue on the ML tree, Figure 2). This topology was also captured by a significant Tajima’s *D* (*D* = −1.88 (95CI: [−1.69–1.84]), P<0.05). It comprised 16 individuals, 10 of the group BEC and 6 of the group BOB. The second group (β) was composed of 27 individuals, 11 from BOB region and 16 from BEC region, with two dominant haplotypes separated by only one mutation in position 126 and two single individual haplotypes (represented in red, Figure 2). The value of the Tajima’s *D* was non-significant (D = −0.80 (95CI: [−1.57–1.77]), P>0.10). The remaining 4 haplotypes (encompassing 7 individuals) are at least at 2 substitutions distant from the nearest haplotype (Figure 2, and Figure S1). The groups α and β were highly supported on the ML tree with branch supports of 76% and 77%, respectively. This separation in two haplogroups α and β was also clearly visualized by the non-metric multi-dimensional scaling (nMDS). Haplotypes from the group α (in blue on Figure 3a) clustered at the right part of the figure, while haplotypes from the group β (in red, Figure 3a) all group together on the left part. Unassigned haplotypes were distributed in between those two groups.

We found no significant correlations between haplogroup membership (α and β) and the sex of the animals (χ^2^ = 0.65, p = 0.42, df = 1), nor with the year of stranding or by-catch (χ^2^ = 4.04, p = 0.77, df = 7) nor the season (χ^2^ = 2.38, p = 0.50, df = 3).

In order to obtain a European-wide picture of the mtDNA structure, we combined our mtDNA dataset with eighty-two previously published mtDNA sequences, all coming from harbour porpoises collected along the East side of the North Atlantic (see Table S3). These data overlap on a 334 bp fragment. Truncating our sequence data set removed 9 polymorphic positions (*i.e.* at position 13 and positions 447 to 569; Table S2), but still defined 10 distinct haplotypes (out of the 15), which matched with those previously published, themselves truncated to the 334 bps overlapping part (Table S4). Similarly, truncating the 82 haplotypes from Genbank eliminated some variable positions and reduced the number of haplotypes identified to 56. The final data set comprised 44 polymorphic sites defining 56 haplotypes coming from Genbank sequences including 10 haplotypes common with our sampling.

The nMDS analysis on this dataset displayed a strongly organized plot depicting a clear geographic structure (Figure 3b). All but two of the haplotypes (S14 et S17, [39]) found in porpoises from the south of the Bay of Biscay clustered on the lower left corner of the plot (orange triangles, Figure 3b). Unambiguously, this cluster also included all the haplotypes of our previously defined group β (red dots, Figure 3b), as well as some haplotypes from individuals from French coasts (purples triangles, Figure 3b). More precisely, this cluster included haplotypes VIA26, VIA27, VIA28, VIA30 and VIA31 [40], found on individuals from the Spanish and French coasts, haplotypes S6, S8, S9, S13, S15 and S16 sampled on French, Portuguese and African coasts [39] together with the truncated form of our haplotypes FrK, FrL, FrM and FrN. Also, this cluster included the haplotype S5, sampled in the North Sea by Tolley & Rosel [39] (green triangle, Figure 3b). The haplotypes from all other porpoises were distributed on the remainder of the plot, and mixed all the animals sampled from geographic positions located to the north of the English Channel (green triangles, Figure 3b). Only two exceptions occurred: haplotypes S14 and S17, sampled respectively along Portuguese and African coasts [39], were clearly visible as 2 orange triangles in the lower right part of the plot (Figure 3b). Also, some individuals stranded or by-caught along the French coasts, especially all novel haplotypes defined in this study and not attributed to the group β were scattered on the rest of the plot (purple triangles, black and blue dots, Figure 3b).

Figure 2b and Figure S1b show the unrooted ML tree and the MJ network for the combined dataset, including the 56 haplotypes. The two analyses depicted a rather shallow phylogeny as observed in previous studies [35], [39], but clearly highlighted the grouping observed with the nMDS analysis. All the haplotypes found in porpoises from African, Portuguese and Spain coasts (orange squares, Figure 2B) grouped together with the haplotypes from group β identified in the present study (red squares, Figure 2B), jointly with some haplotypes coming from French samples of previous studies [39], [40]. Branch support for this group was particularly high (84%).

### Microsatellite Variation Analysis

Seven microsatellite loci, previously identified by Rosel *et al.* [41] and genotyped here were all polymorphic and showed between 7 and 15 alleles in our samples (Table 1, and Table S5). Allelic richness varied from 5.81 (PPHO131) to 10.56 (PPHO130), and observed and expected heterozygosity ranged from *H_o_* = 0.457 to *H_o_* = 0.913 and *H_e_* = 0.670 to *H_e_* = 0.885. No evidence of linkage disequilibrium was found in any pairwise locus comparison (p>0.05 for all pair-wise comparisons). Allelic frequencies displayed no significant departure from Hardy-Weinberg expectations in the global sample (*F_IS_* = 0.01 (95CI:[−0.08–0.12]), p = 0.314), nor for each sub-grouping we considered based on mitochondrial haplogroup (haplogroup α, *F_IS_* = 0.08 (95CI:[−0.05–0.21]), p = 0.061 and β, *F_IS_* = −0.02 (95CI:[−0.11–0.08]), p = 0.743), nor for sub-grouping based on geography (BEC, *F_IS_* = 0.02 (95CI:[−0.10–0.17]), p = 0.304 and BOB, *F_IS_* = −0.01 (95CI:[−0.07–0.07]),p = 0.596). However, one locus (PPHO102) displayed a significant deficit of heterozygosity in the global sample (*F_IS_* = 0.321, p<0.05) and within the group BEC (*F_IS_* = 0.388, p<0.05). All other *F_IS_* values were non-significant (Table S5). In agreement with the absence of departure from HW expectations, we did not detect any significant differences in allelic frequencies between the two geographic groups (BEC *versus* BOB, *F_ST_* = 0.01 (95CI: [−0.01–0.03]), p = 0.065) nor between the two mtDNA haplogroups (α *versus* β, *F_ST_* = −0.01 (95CI:[−0.02–0.00]), p = 0.929).

**Table 1:** Summary statistics for the 7 microsatellite loci analyzed.

All samples												
Locus	N	nA		range		*He*		*Ho*	A		*Fis*	
PPHO110	46	9		124–146		0.795		0.891	6.35		−0.122	
PPHO130	45	15		113–143		0.834		0.844	10.56		−0.013	
PPHO137	45	15		159–189		0.885		0.756	10.24		0.148	
PPHO102	46	10		176–198		0.670		0.457	7.48		0.321*	
PPHO142	46	15		174–208		0.789		0.826	9.60		−0.048	
PPHO104	46	13		193–233		0.860		0.891	9.30		−0.037	
PPHO131	46	7		110–130		0.809		0.913	5.81		−0.130	
All	46	12				0.806		0.797	8.48		0.011	
	**Group BEC (Britanny and English Channel)**						**Group BOB (Bay Of Biscay)**					
**Locus**	**A**		**npA**		***He***		**A**			**npA**		***He***
PPHO110	5.52		0.04		0.783		7.71			3.11		0.822
PPHO130	10.03		2.44		0.808		10.53			2.97		0.859
PPHO137	8.39		1.66		0.825		11.55			5.41		0.892
PPHO102	7.59		1.87		0.686		7.34			1.37		0.654
PPHO142	9.95		2.38		0.753		10.03			2.61		0.836
PPHO104	8.97		1.55		0.826		9.91			2.50		0.895
PPHO131	5.54		0.04		0.814		6.38			1.21		0.818
All	8.00		1.42		0.785		9.06			2.74		0.825

Data are expressed for each locus and as the average of all loci for all samples, and for arbitrary geographic groups BEC and BOB.

N = sample size, nA = number of alleles, range = range of allele sizes in bp, *He* = non biased expected heterozygosity, *Ho* = observed heterozygosity, *A* = allelic richness (estimated for a sample size of 14 individuals), *npA* = number of private alleles (estimated for a sample size of 19 individuals), *F_is_*_ = _value *F_is_* calculated after Weir and Cockerham. Asterisks mark significant departure from HWE after the Bonferroni correction (*: p<0.05).

The analysis of allelic richness, private alleles and excepted heterozygosity are presented in Table 1 for the BOB and the BEC groups. Group BOB displayed higher values of private alleles than group BEC, as well as higher levels of heterozygosity and allelic richness (Wilcoxon signed ranked (WSR) test, p = 0.05). The groups α and β did not display such difference (WSR test, p>0.05).

We further investigated potential population subdivision by conducting a Bayesian clustering analysis using the program Structure V2.3 [44]–[46]. All the model settings we tested, *i.e.* with or without admixture and using the standard or the *locprior* model, returned comparable results: the data did not contain any evidence of population subdivision. The number of groups (K) that best explained the data was K = 1 with a posterior probability for this value of p>0.99 (Figure S2). Therefore, the analysis of microsatellite variation did not reveal any evidence of population subdivision within our global sample, in contrast to the results obtained based on the mtDNA control region sequence polymorphisms.

To ensure that such lack of significant differentiation is not the result from a low power, we evaluated the statistical power that can be achieved using our microsatellite dataset, representative of the animal sampling and of the loci number and polymorphisms, using Powsim [47]. Fontaine *et al.* [16] estimated effective population sizes for both Iberian and northern Bay of Biscay harbour porpoise populations as, respectively, n = 79 and n = 353, and a splitting time between the two populations of at least 35 generations. On this basis, we used the most stringent values, an effective population size of 353 individuals and a number of generations (t) set to 35, and the genetic differentiation was quantified as *F_ST_* = 0.049. A higher number of generations, or a lower population size such as the one calculated for the Iberian population would have lead to a higher genetic differentiation. Fisher’s exact test and Chi-square estimated that, using our sampling (n**_α_** = 14 and n**_β_** = 21), such level of genetic differentiation would have been detected, if existing, in all cases (100% probability).

Rather than being the result of a low statistical power due to small sample size, the lack of genetic differentiation detected by microsatellite polymorphisms is instead likely to reflect actual genetic admixture between harbour porpoises along the French Atlantic coast.

## Discussion

Duguy observed that, having once been one of the most common cetacean species, the harbour porpoise had become rare along the French Atlantic coasts [9]. It is only since the mid-1990s that a recovery of the species has been confirmed in this area [8]. It was believed that the abundance recovery was simply related to a general southward shift of the species detected in European waters by the two SCANS campaigns [3], [5] and by local studies on European mainland coasts north to the English Channel [6], [11]–[13]. However, biological interactions between Iberian and French harbour porpoises have long been hypothesized [48]. In fact, although recent genetic studies identified two distinct populations surrounding the Bay of Biscay, they also detected northward migrants from the Iberian population to the North East Atlantic [15], [16]. Thus, the population structure of the harbour porpoise along the French coasts needed to be elucidated to properly understand the change in the species’ distribution.

### Dual Geographic Origin of French Harbour Porpoises

Both the haplotype networks and ML phylogenetic trees highlighted the existence of two mitochondrial haplogroups in the harbour porpoises found along the French Atlantic coasts. The nMDS analysis provided a particularly clear-cut picture of the genetic distinction between groups of harbour porpoises both at a local scale in the Bay of Biscay and at the eastern North Atlantic scale, when we compared our new data with those previously published [31], [32], [36], [39], [40]. One of the haplogroups (*i.e.* β) clustered with the haplotypes found in Iberian and African harbour porpoises, together with some French individuals previously analysed [39], [40]. This “South cluster” appeared very clearly on the ML tree, and contained only one exception, an individual sampled in the North Sea [39]. Only two haplotypes sampled from Iberian and African harbour porpoises by Tolley & Rosel [39] are missing from the “South cluster”. The second haplogroup (*i.e.,* α) corresponded to harbour porpoises originating from North East Atlantic, including the North Sea, the English Channel and the French coasts [31], [32], [39], [40]. Haplotypes previously identified for French harbour porpoises could either belong to one of the two groups.

The mtDNA data thus clearly revealed that harbour porpoises found along the French Atlantic coasts display a dual genetic origin. This result is consistent with previous observations of Tolley & Rosel [39] who documented genetic structuring in the harbour porpoise population along the European coasts. However, the nMDS analysis performed in this study provided an even higher resolution. The two genetic haplogroups defined on the basis of mtDNA variation thus have a geographic explanation, and enabled us to distinguish between the African-Iberian population and individuals originating from North East Atlantic waters.

Accordingly, the co-existence of these two haplogroups along the French coasts shows that some individuals migrated southward from the northern waters, or were the offspring of such migrants. Their movement can partially explain the recovery of the harbour porpoise along the coasts of France, and certainly correspond with the more global southward shift in the species distribution in the eastern North Atlantic waters [5]. However, it is also clear that some harbour porpoises originated from the Iberian population and crossed the putative environmental barrier to dispersal presented by the Capbreton canyon and migrated northwards to the Bay of Biscay and further north to the coasts of Brittany. They, or their offspring, account for more than the half of the harbour porpoises of our sampling. This phenomenon is clearly recent, as suggested by the increased observations of harbour porpoises along the French coasts in the last ten years after their quasi-disappearance from the same geographic area [8], [9].

### The Lack of Genetic Structure in Autosomal Microsatellite Loci

The analysis of microsatellite polymorphism provided a distinct perspective from the mtDNA one. The seven loci analyzed were highly polymorphic, but they did not reveal any evidence of genetic subdivision in our sampling. We detected no departure from Hardy-Weinberg expectations and none of our analyses based on allelic frequency or on the Bayesian clustering detected any population subdivisions. Even the new algorithm of Structure 2.3.3 (the “*locprior”* model, [46]) designed to detect weak genetic structure did not help in recovering any population subdivision.

Evaluation of the statistical power of our analysis using the simulation-based procedure of Powsim showed that the lack of genetic structure detection is not caused by low statistical power. Indeed, the statistical power of detecting a significant *F_ST_* value, given the effective population sizes of the Iberian population and the northern Biscayan population estimated in [16], the number of loci and the sample size we have analysed here, reached about 100%. This indicates that only biological process can explain this lack of population structure at autosomal loci. It is to note that the simulation of the genetic drift between the Iberian and the population further north using Powsim led to *F_ST_* values remarkably close to the observed values, calculated by Fontaine et al. [15], still reinforcing the significance of this evaluation.

The fact that microsatellite allele frequencies do not depart from HW expectations clearly suggests that, at this geographic scale, harbour porpoises in the Bay of Biscay and along the coasts of Brittany behave as a randomly mating unit. On the other hand, mitochondrial data show that harbour porpoises in this area are a mixture from the two populations surrounding the Bay of Biscay. One hypothesis is that admixture between two genetically distinct populations can explain such combination of results. Hardy Weinberg equilibrium can be restored after just one generation, if the populations truly behave as a global random mating unit [49]. This could thus explain why we observed no departure from HW expectations along the Atlantic coasts of France. Female harbour porpoises reach sexual maturity at 3–4 years of age [17], and thus only a few years are needed to generate hybrid offspring. Therefore, this would imply that a significant proportion of the animals sampled constitute admixed individuals derived from the two genetically distinct populations previously identified along the Iberian coasts and in the North East Atlantic [15]. However, quantifying the exact proportions of admixture will require more detailed sampling of source populations.

The only signal of genetic structure that we detected in the nuclear markers was the significantly higher value of allelic richness and private allelic richness in samples coming from the Bay of Biscay (group BOB) compared to those of the Brittany and English Channel (group BEC). As these groups were defined on a geographic basis, this result could be explained by the isolation by distance pattern demonstrated for the harbour porpoise in North East Atlantic waters [15]. But the absence of significant differences in allelic frequencies between the two groups, combined with the relatively small distance and absence of any natural barrier between the two geographic areas, leads us to believe that, if it does exist, this genetic difference is very weak. The proportion of animals coming from the Iberian waters should be higher in the group BOB, closer to the Capbreton canyon than the group BEC, thus leading to this higher genetic diversity.

### Implications in Terms of Conservation

The French Atlantic coast clearly appears to be an area of contact and probably admixture between two previously separated populations of harbour porpoises. Harbour porpoises had almost totally disappeared in this area by the mid 1990s, but they have made, and continue to make, a strong recovery, with increasing number of sightings and strandings [8]. This suggests that habitat conditions are becoming more suitable to sustain the return of harbour porpoises along the French coast. Attention should be paid to this specific area in the future both in terms of conservation and further study. As recommended [50], the by-catch of harbour porpoises will have to be carefully evaluated, and campaigns to number individuals will have to be planned in order to evaluate the percentage of the population impacted by by-catch. The recent creation of the first French Marine Park in the Iroise Sea (official site: http://www.parc-marin-iroise.gouv.fr) will obviously help in this required conservation effort. A program of scientific studies, named “INPECMAM”, has been defined, with the aim of following the small cetacean by-catch events in the Iroise Sea, and the harbour porpoise will be an important component of this study.

### What are the Possible causes of the Harbour Porpoise Shifts?

Besides being informative for conservation efforts, our results underline the value of studying the changes in distribution of the harbour porpoise in European waters, with a special focus on the French coasts. The Bay of Biscay and the waters off the coasts of Brittany deserve special attention, as this area represents a well-known biogeographic transition zone between temperate species and subtropical species [51]. The impact of climate change could hence be more visible here [52]. The harbour porpoise was almost absent from this area until recently, but a genetic signal of migration between the two populations surrounding it was already detected previously [16]. Our study strongly suggests that most of these migration events could have occurred in the last few years. Shifts in harbour porpoise distribution are thus ongoing in European waters, and the French coast is particularly significant because there are two concomitant shifts occurring, one southward and the other northward. The geographic limits of the northward migration of the Iberian population will have to be determined in the coming years, as well as the limits of the southward shift of the North East Atlantic population.

Habitat changes, especially ones potentially affecting the food availability, are suspected to be the cause for marine predator displacements. Such changes would be significant for harbour porpoises, because they have only limited energy storage capacity [18]. Climate change has been shown to affect fish distributions [52], [53], but except for some specific cases, its effects on cetaceans, often highly mobile, have been difficult to study [19], [29], [54]. Fontaine *et al.* [16] hypothesized that the northward migration of the Iberian harbour porpoise population could be interpreted as a response to ocean warming, as might be expected for a temperate predator in the Northern hemisphere. Our results may well support this theory.

The complementary use of nuclear and mitochondrial markers has enabled us to uncover that the harbour porpoises found along the coasts of France have a dual origin, and that the two populations are currently hybridizing. The coasts of France therefore appear to be an area of major significance for Atlantic harbour porpoises, and may provide a key to understanding the range shift of a marine apex predator in response to a changing environment.

## Materials and Methods

### DNA Extraction and PCR-based Sex Determination

Samples were derived from different organs (skin, blubber, muscle, kidney and liver) and all kept either at room temperature in 95° ethanol or frozen at −20°C. Total genomic DNA was extracted from all types of samples using the DNeasy Blood and Tissue Kit (Qiagen) following the manufacturer recommendations. The quality of the extracted DNA was estimated by agarose gel electrophoresis and concentrations were determined using a Nanodrop 1000 (Thermo Scientific). The sex of each animal was determined as described [55], [56].

### Amplification, Sequencing and Analysis of the Mitochondrial DNA Control Region

A fragment of 623 bp, from position 15375 to 15997 on the *P. phocoena* complete mitochondrial genome sequence (Genbank ref. AJ554063.1), including the mitochondrial control region, was amplified using two primers: mcrf (5′-acctcggtcttgtaaacc-3′) and mcrr (5′-accaaatgaatgaaatctcag-3′), derived from primers L15928 and HOOO34 [33], [57]. The polymerase chain reactions (PCR) were carried out in 50 µl of final volume containing around 50 ng of genomic DNA, 1 µM of each primer in the Hotgoldstar master mix x1 (Eurogentec) with a final concentration of MgCl_2_ of 2.5 mM. After an initial denaturation step of 10 min at 95°C, the cycling parameters were: 5 cycles of 95°C for 30 s, 46°C for 30 s and 72°C for 60 s each, followed by 35 cycles consisting 95°C for 30 s, 53°C for 30 s and 72°C for 60 s. Reactions were ended by a final extension step of 10 min at 72°C.

The PCR products were purified using the Quick Clean 5M PCR purification Kit (Genscript) and sequenced on an ABI3730XL sequencer by Macrogen (Korea), in presence of one of the primers used for DNA amplification. Each haplotype was sequenced on both directions, *i.e.* in forward and reverse sense, at least one time. Results of the sequence reactions were analyzed using the Sequence Scanner software (Applied Biosystem). Consensus sequences and alignments were produced using Bioedit [58].

In addition to these new data, a total of eighty two sequences of mitochondrial control region of European and African harbour porpoises were found in Genbank by a key-word search, using “D-Loop” or “Control region” restricted to the *Phocoena phocoena* species, and then excluding Pacific, East Atlantic and Black Sea samples (Table S3). All the data overlapped for a 334 bps length fragment of the D-loop region starting at the position 15477 of the complete mitochondrial genome (Genbank ref. AJ554063.1). All the sequences including the new ones uncovered in this study were truncated to this common 334 bps part to produce a second dataset combining local data with European ones on which the analyses were replicated (Table S6).

### Data Analyses

For population genetic analysis, we used Arlequin 3.11 [59] in order to identify the different haplotypes and DnaSP V.5.10 to calculate haplotype and nucleotide diversities and Tajima’s *D* statistics [60], [61]. Phylogenetic relationships among haplotypes were depicted using a median joining network of haplotypes using Network 4.6 (www.fluxus-engineering.com). We also used a maximum-likelihood (ML) approach to construct the phylogenetic trees using an online phylogeny pipeline [62]. Sequences were aligned using MUSCLE [63], a ML tree was built using PhyML with a HKY85 model of sequence evolution and the gamma correction [64]. The tree was drawn using TreeDyn [65]. Branch supports were tested using the approximate likelihood-ratio method [66]. We also tested a parsimony approach (TNT, [67]) and neighbor-joining distance-based methods (BIONJ, [68]) to check for the consistency of the results.

We used a multi-dimensional scaling (MDS) approach in order to graphically represent genetic distances between haplotypes. The model used is an ordinal (non metric) nMDS using monotone regression and rank images. This method display each haplotype sequence in a n-dimensional geometric space so as to respect as much as possible the rank order of the calculated genetic distances between each pair of sequences. We computed the distance matrix using DNAdist [69] the F84 models and similarities table parameters being both tested. Distances matrix or 1-similarity matrix were then analyzed by nMDS using Statistica (Statsoft, 2005).

Genetic differentiation between subpopulations was tested at both the haplotype frequency level using the *H_ST_* statistics [42] and at the nucleotidic level using the *Snn* statistic [43], both implemented in the DnaSP v5.10 software [60].

### Microsatellite Analysis

We screened 7 nuclear microsatellite loci for 46 harbour porpoises. We first established new reaction conditions based on the published sequences of microsatellite-containing loci [41] and a three primers-reaction approach that use an universal primer linked to the fluorochrome and a couple of primers specific to the locus, one of which is extended at its 5′-end by the universal primer sequence [70]. PCR were carried out in presence of these three primers, with a molar ratio between the 3 primers of 1/100/100 (specific-tailed, reverse, universal), in order to allow a progressive incorporation of the universal primer in new amplicons, thus labeling PCR products after only few cycles. This protocol facilitates use of one universal primer per each fluorescent dye, instead of linking the dye to one of the locus-specific primer. All primers were designed on the basis of the published sequences with the help of the OligoAnalyser tool (V. 3.1 on line at http://eu.idtdna.com). The Table S7 provides the Genbank references of the published loci [41], the sequences of the specific primers for each locus, and the sequences of the two universal primers used in this study.

PCR reactions (25 µl of final volumes) contained around 10 ng of genomic DNA, 10 pmole of universal and reverse primers, 0.1 pmole of specific-tailed primer, 2.5 mM of MgCl*2*, 200 µM of each dNTP and 1 unit of TAQ polymerase (Eurogentec, Belgium) in the standard reaction buffer. Cycling profiles consisted in an initial denaturation step at 94°C for 10 min, followed by 35 cycles of 94°C for 30 s, 30 s at 55°C and 72°C for 30 s, and ended with a final extension step of 15 min at 72°C. One µl of each reaction was diluted in water, mixed with 0.25 µl of GENESCAN 500 ROX (Applied Biosystem), and analyzed on an Applied Biosystems 3130 Genetic Analyzer after a 5 min. denaturation at 95°C.

The Peak Scanner software (Applied Biosystems) was used to determine the quality of the reactions and the lengths of the amplified fragments. Allele sizes were then defined on the basis of these results, genotyping values were grouped in an Excel spreadsheet (Microsoft), and converted to the required formats for further analysis using PGDSpider [71].

Genetic polymorphism at each locus was quantified using allelic richness (A) and private allelic richness (pAr) measures calculated using ADZE [72], observed and unbiased expected heterozygosities (*Ho* and *He*) and fixation indexes (*F_IS_*) were calculated using FSTAT 2.9.3.2 [73]. Departures from Hardy-Weinberg expectations were tested using exact tests with the sequential Bonferroni correction for multiple comparisons [74]. Linkage disequilibrium among loci was tested using a permutation test (10^5^) implemented in FSTAT 2.9.3.2 [73] Differences in allelic frequencies between groups of porpoises were tested using exact tests implemented in GENEPOP 4.0 [75] and quantified using the Weir and Cockerham estimator of *F_ST_* [76].

We assessed the statistical power of our microsatellite data set using Powsim [47]. Powsim simulates genetic drift between two independent populations of given sizes and for a specified number of generations. The effective population sizes (*Ne*) and the number of generations (t) were taken from Fontaine *et al.* [16], and allowed Powsim to simulate genetic drifts between the Iberian and the northern bay of Biscay populations since the time of splitting, and to estimate the resulting genetic differentiation quantified as *F_ST_*. Present numbers of samples were then used to calculate the proportion of significant outcomes among 1000 repetitions using chi-square and Fisher’s exact tests, leading to an evaluation of the relevance of the sample sizes and genetic markers used in this study to detect the expected genetic differentiation.

We further investigated the population structure using the Bayesian model-based clustering algorithm implemented in Structure 2.3.3 [44]–[46]. This analysis partitions multilocus genotypes into clusters, while minimizing departure from Hardy Weinberg and linkage equilibrium (HWLE) among loci, and estimates the ancestry proportions to the different populations. We conducted the analyses on the multilocus microsatellite genotypic dataset. The analysis was performed using the “standard model” of population admixture and allele frequencies correlated among populations, and also with a no admixture model. A second series of analysis was performed using the new « *locprior* » model recently developed by Hubisz *et al.* [46] designed to detect weak population structure by making explicit use of sampling location information. To that aim, we made use of an *a priori* assumption that porpoises from the genetic haplogroups described in this manuscript came from two distinct populations by modifying the prior on individual origin in the model. Other settings for the model simulations were as follow. We conducted a series of independent runs with different proposals for the number of clusters (K), testing all values from 1 to 5. Each run used 500,000 iterations after a burn-in of 50,000 iterations. To ensure convergence of the Markov Chain Monte Carlo (MCMC), we performed 5 independent replicates for each value of K. The number of clusters that best explains the data was tested by computing the posterior probability of the data for a given number of clusters tested, P(X|K) and by computing the rate of change of this value as K is increased [77].

### Ethics Statements

The study was entirely based on samples collected from cetacean carcasses found stranded or accidently by-caught along the French coasts and did not involve observation or experimentation on captive animals by any mean.

The University of La Rochelle is the institution permanently in charge of running the French marine mammal stranding network under the decree of 10 November 2010, jointly taken by the Ministery in charge of the Environment and the Ministery in charge of Fisheries, regarding the use of biological data and samples collected on stranded marine mammals for scientific research and monitoring purposes.

## Supporting Information

Figure S1 Mitochondrial haplotype networks. **A.** Haplotype network depicting the relationships between the 15 harbour porpoises mtDNA control region haplotypes determined in this study. **B.** Haplotype network of the 56 truncated haplotypes of mtDNA control region of harbour porpoise determined in this study and in previous ones.
(TIF)

Figure S2 Mean probabilities [LnPr(X|K)] of the data as a function of the fixed number of clusters (K).
(DOC)

Table S1 List of the 52 harbour porpoises sampled.
(DOCX)

Table S2 Variable sites in the 15 mtDNA control region haplotypes defined in this study.
(DOCX)

Table S3 List of the mtDNA control regions haplotypes previously determined by other authors on harbour porpoises and used in this study.
(DOCX)

Table S4 Haplotype data sets analyzed.
(DOCX)

Table S5 Summary statistics for the 7 microsatellite loci analyzed and for each group of samples.
(DOCX)

Table S6 Correspondances between truncated haplotypes and their coding on ML phylogenetic tree and on haplotype networks.
(DOCX)

Table S7 Primer sequences for the seven microsatellites loci analyzed in this study.
(DOCX)

## References

[1] Gaskin DE (1984) The harbour porpoise *Phocoena phocoena* (L.): Regional populations, status, and information on direct and indirect catches. Rep Int Whal Comm 34: 569–586.

[2] Read A (1999) Harbour porpoise - *Phocoena phocoena* (Linnaeus, 1758). In: Ridgway S, Harrison S, editors. Handbook of marine mammals: the second book of dolphins and porpoises. San Diego, CA: Academic Press, Vol. 6: 323–356.

[3] Hammond P, Berggren P, Benke H, Borchers D, Collet A, et al (2002) Abundance of harbour porpoise and other cetaceans in the North Sea and adjacent waters. J App Ecol 39: 361–376.

[4] Kiska S, Hassani S, Pezeril S (2004) Distribution and status of small cetaceans along the French Channel coasts: using opportunistic records for a preliminary assessment. Lutra 47: 33–46.

[5] Anonymous (2006) Small cetaceans in the European Atlantic and North Sea (SCANS-II). LIFE Project No. LIFE04NAT/GB/000245. University of St Andrews, Fife, Scotland, UK. Available at http://biologyst-andacuk/scans2/inner-finalReporthtml: 1–55.

[6] Thomsen F, Laczny M, Piper W (2006) A recovery of harbour porpoises (*Phocoena phocoena*) in the southern North Sea? A case study off Eastern Frisia, Germany. Helgol Mar Res 60: 189–195 doi:10.1007/s10152–006–0021-z.

[7] Van Der Meij S, Camphuysen K (2006) The distribution and diversity of whales and dolphins (Cetacea) in the southern North Sea: 1970–2005. Lutra 49: 3–28.

[8] Jung JL, Stéphan E, Louis M, Alfonsi E, Liret C, et al (2009) Harbour porpoises (*Phocoena phocoena*) in north-western France: aerial survey, opportunistic sightings and strandings monitoring. J Mar Biol Ass 89: 1045–1050 doi:10.1017/S0025315409000307.

[9] Duguy R (1977) Notes on the small cetaceans off the coasts of France. Rep Int Whal Comm 27: 500–501.

[10] Reijnders P (1992) Harbour porpoises *Phocoena phocoena* in the North Sea: Numerical responses to changes in environmental conditions. Neth J Aquat Ecol 26: 75–85.

[11] Camphuysen K (2004) The return of the harbour porpoise (*Phocoena phocoena*) in Dutch coastal waters. Lutra 47: 113–122.

[12] Siebert U, Gilles A, Lucke K, Ludwig M, Benke H, et al (2006) A decade of harbour porpoise occurrence in German waters - Analyses of aerial surveys, incidental sightings and strandings. J Sea Res 56: 65–80 doi:10.1016/j.seares.2006.01.003.

[13] Pikesley S, Witt M, Hardy T, Loveridge J, et al.. (2011) Cetacean sightings and strandings: evidence for spatial and temporal trends? J Mar Biol Ass DOI:10.1017/S0025315411000464.

[14] Lockyer C (2007) All creatures great and smaller: a study in cetacean life history energetics. J Mar Biol Ass 87: 1035–1045 doi:10.1017/S0025315407054720.

[15] Fontaine MC, Baird SJ, Piry S, Ray N, Tolley KA, et al (2007) Rise of oceanographic barriers in continuous populations of a cetacean: the genetic structure of harbour porpoises in Old World waters. BMC Biol 5: 30 doi:10.1186/1741-7007-5-30. 17651495 10.1186/1741-7007-5-30 PMC1971045

[16] Fontaine MC, Tolley KA, Michaux JR, Birkun A, Ferreira M, et al (2010) Genetic and historic evidence for climate-driven population fragmentation in a top cetacean predator: the harbour porpoises in European water. P Roy Soc Lond B 277: 2829–2837 doi:10.1098/rspb.2010.0412. 10.1098/rspb.2010.0412 PMC2981983 20444724

[17] Lockyer C (2003) Harbour porpoises (*Phocoena phocoena*) in the North Atlantic: Biological parameters. NAMMCO Sci Publ 5: 71–90.

[18] Koopman H, Pabst D, McLellan W, Dillaman R, Read A (2002) Changes in Blubber Distribution and Morphology Associated with Starvation in the Harbor Porpoise (*Phocoena phocoena*): Evidence for Regional Differences in Blubber Structure and Function. Physiol Biochem Zool 75: 498–512. 12529851 10.1086/342799

[19] MacLeod CD, Santos MB, Reid RJ, Scott BE, Pierce GJ (2007) Linking sandeel consumption and the likelihood of starvation in harbour porpoises in the Scottish North Sea: could climate change mean more starving porpoises? Biol Letters 3: 185–188 doi:10.1098/rsbl.2006.0588. 10.1098/rsbl.2006.0588 PMC2375924 17251125

[20] Santos M, Pierce G, Learmonth J, Reid R, Ross H, et al (2004) Variability in the diet of harbor porpoises (*phocoena phocoena*) in scottish waters 1992–2003. Mar Mammal Sci 20: 1–27.

[21] Johnston DW, Westgate AJ, Read AJ (2005) Effects of fine-scale oceanographic features on the distribution and movements of harbour porpoises *Phocoena phocoena* in the Bay of Fundy. Mar Ecol Prog Ser 295: 279–293.

[22] Santos MB, Pierce GJ (2003) The diet of harbour porpoise (*phocoena phocoena*) in the northeast atlantic. Oceanogr Mar Biol: Ann Rev 41: 335–390.

[23] Herr H, Fock HO, Siebert U (2009) Spatio-temporal associations between harbour porpoise *Phocoena phocoena* and specific fisheries in the German Bight. Biol Conserv 142: 2962–2972 doi:10.1016/j.biocon.2009.07.025.

[24] Tregenza N, Berrow S, Hammond P, Leaper R (1997) Harbour porpoise (*Phocoena phocoena* L.) by-catch in set gillnets in the Celtic Sea. ICES J Mar Sci 54: 896–904.

[25] Northridge S, Hammond P (1999) Estimation of porpoise mortality in UK gill and tangle net fisheries in the North Sea and west of Scotland. Paper presented to the Scientific Committee of the international whaling Commission Paper SC/51/SM42.: Grenada, WL.

[26] Berggren P, Wade P, Carlström J, Read A (2002) Potential limits to anthropogenic mortality for harbour porpoises in the Baltic region. Biol Conserv 103: 313–322.

[27] Stenson G (2003) Harbour porpoise (*Phocoena phocoena*) in the North Atlantic: Abundance, removals, and sustainability of removals. In: Haug T, Desportes G, Vikingsson G, Witting L, editors. Harbour porpoises in the North Atlantic. NAMMCO Sci Pub, Vol. 5: 271–302.

[28] Gambaiani DD, Mayol P, Isaac SJ, Simmonds MP (2008) Potential impacts of climate change and greenhouse gas emissions on Mediterranean marine ecosystems and cetaceans. J Mar Biol Ass 89: 179–201 doi:10.1017/S0025315408002476.

[29] Simmonds MP, Eliott WJ (2009) Climate change and cetaceans: concerns and recent developments. J Mar Biol Ass 89: 203–210 doi:10.1017/S0025315408003196.

[30] Fontaine MC, Snirc A, Frantzis A, Koutrakis E, Öztürk B, Öztürk AA, Austerlitz F (2012) History of expansion and anthropogenic collapse in a top marine predator of the Black Sea estimated from genetic data. Proc Natl Acad Sci USA. *In press*. 10.1073/pnas.1201258109 PMC3458354 22949646

[31] Walton MJ (1997) Population structure of harbour porpoises *Phocoena phocoena* in the seas around the UK and adjacent waters. Proc R Soc Lond B 264: 89–94. 10.1098/rspb.1997.0013 PMC1688231 9061964

[32] Tiedemann R, Harder J, Gmeiner C, Haase E (1996) Mitochondrial DNA sequence patterns of Harbour porpoises (*Phocoena phocoena*) from the North and the Baltic sea. Zeit Saugetierkunde 61: 104–111.

[33] Rosel PE, Dizon AE, Haygood MG (1995) Variability of the mitochondrial control region in populations of the harbour porpoise, *Phocoena phocoena*, on interoceanic and regional scales. Canadian J Fish Aquat Sci 52: 1210–1219.

[34] Chivers SJ, Dizon AE, Gearin PJ, Robertson KM (2002) Small-scale population structure of eastern North Pacific harbour porpoises (*Phocoena phocoena*) indicated by molecular genetic analyses. J Cetacean Res Manage 4: 111–122.

[35] Tolley KA, Rosel PE, Walton M, Bjørge A, Øien N (2001) Genetic population structure of harbour porpoises (*Phocoena phocoena*) in the North Sea and Norvegian waters. J Cetacean Res Manage 1: 265–274.

[36] Tolley KA, Vikingsson GA, Rosel PE (2001) Mitochondrial DNA sequence variation and phylogeographic patterns in harbour porpoises (*Phocoena phocoena*) from the North Atlantic. Conserv Genet 2: 349–361.

[37] Andersen LW, Ruzzante DE, Walton M, Berggren P, Bjørge A, et al (2001) Conservation genetics of harbour porpoises, *Phocoena phocoena*, in eastern and central North Atlantic. Conserv Genet 2: 309–324.

[38] Wiemann A, Andersen LW, Berggren P, Siebert U, Benke H, et al (2010) Mitochondrial Control Region and microsatellite analyses on harbour porpoise (*Phocoena phocoena*) unravel population differentiation in the Baltic Sea and adjacent waters. Conserv Genet 11: 195–211 doi:10.1007/s10592–009–0023-x.

[39] Tolley KA, Rosel PE (2006) Population structure and historical demography of eastern North Atlantic harbour porpoises inferred through mtDNA sequences. Mar Ecol Prog Ser 327: 297–308.

[40] Viaud-Martinez KA, Vergara MM, Gol’din PE, Ridoux V, Ozturk AA, et al (2007) Morphological and genetic differentiation of the Black Sea harbour porpoise *Phocoena phocoena* . Mar Ecol Prog Ser 338: 281–294.

[41] Rosel PE, France SC, Wang JY, Kocher TD (1999) Genetic structure of harbour porpoise *Phocoena phocoena* populations in the northwest Atlantic based on mitochondrial and nuclear markers. Mol Ecol 8: S41–S54. 10703550 10.1046/j.1365-294x.1999.00758.x

[42] Hudson RD, Boos DB, Kaplan NL (1998) A Statistical Test for Detecting Geographic Subdivision. Mol Biol Evol 9: 138–151. 10.1093/oxfordjournals.molbev.a040703 1552836

[43] Hudson RD (2000) A New Statistic for Detecting Genetic Differentiation. Genetics 155: 2011–2014. 10924493 10.1093/genetics/155.4.2011 PMC1461195

[44] Pritchard JK, Stephens M, Donnelly P (2000) Inference of Population Structure Using Multilocus Genotype Data. Genetics 155: 945–959. 10835412 10.1093/genetics/155.2.945 PMC1461096

[45] Falush D, Stephens M, Pritchard JK (2003) Inference of Population Structure Using Multilocus Genotype Data: Linked Loci and Correlated Allele Frequencies. Genetics 164: 1567–1587. 12930761 10.1093/genetics/164.4.1567 PMC1462648

[46] Hubisz MJ, Falush D, Stephens M, Pritchard JK (2009) Inferring weak population structure with the assistance of sample group information. Mol Ecol Res 9: 1322–1332 doi:10.1111/j.1755–0998.2009.02591.x. 10.1111/j.1755-0998.2009.02591.x PMC3518025 21564903

[47] Ryman N, Palm S (2006) Powsim: a computer program for assessing statistical power when testing for genetic differentiation. Mol Ecol 6: 600–602 doi:10.1111/j.1365–294X.2006.01378.x. 10.1046/j.0962-1083.2001.01345.x 11703649

[48] Donovan GP, Bjørge A (1995) Harbour porpoises in the North Atlantic. In: Rep Int Whal Comm, Vol. Special Issue Bjørge A, Donovan GP, editors. Biology of the Phocoenids. 16: 3–25.

[49] Hartl DL, Clark AG (2007) Principles of Population Genetics. Sunderland, MA: Sinauer and Associates. 652 p.

[50] Reijnders PJH, Donovan GP, Bjørge A, Koch K-H, Eisfeld S, et al. (2009) ASCOBANS Conservation Plan for Harbour Porpoises (*Phocoena phocoena* L.) in the North Sea. Report presented to the 16th Advisory Committee meeting 16, Bruges, Belgium, 20–24 April 2009, AC16/Doc.21.

[51] Southward AJ, Hawkins SJ, Burrows MT (1995) Seventy years’ observations of changes in distribution and abundance of zooplankton and intertidal organisms in the western English Channel in relation to rising sea temperature. J Therm Biol 20: 127–155.

[52] Poulard J, Blanchard F (2005) The impact of climate change on the fish community structure of the eastern continental shelf of the Bay of Biscay. ICES J Mar Sci 62: 1436–1443 doi:10.1016/j.icesjms.2005.04.017.

[53] Perry AL (2005) Climate Change and Distribution Shifts in Marine Fishes. Science 308: 1912–1915 doi:10.1126/science.1111322. 15890845 10.1126/science.1111322

[54] Palacios DM, Salazar SK, Vargas FH (2011) Galápagos Marine Vertebrates: Responses to Environmental Variability and Potential Impacts of Climate Change. In: Larrea I, Di Carlo G, editors. Climate Change Vulnerability Assessment of the Galápagos Islands. USA: WWF and Conservation International. 69–80.

[55] Rosel P (2003) PCR-based sex determination in Odontocete cetaceans. Conserv Genet 4: 647–649.

[56] Jayasankar P, Anoop B, Rajagopalan M (2008) PCR-based sex determination of cetaceans and dugong from the Indian seas. Current Sci 94: 1513–1516.

[57] Kocher TD, Thomas WK, Meyer A, Edwards SV, Pââbo S, et al (1989) Dynamics of mitochondrial DNA evolution in animals: Amplification and sequencing with conserved primers. Proc Natl Acad Sci USA 86: 6196–6200. 2762322 10.1073/pnas.86.16.6196 PMC297804

[58] Hall TA (1999) BioEdit: a user-friendly biological sequence alignment editor and analysis program for Windows 95/98/NT. Nucleic Acids Symp Ser 41: 95–98.

[59] Excoffier L, Laval G, Schneider S (2005) Arlequin (version 3.0): An integrated software package for population genetics data analysis. Evolutionary Bioinformatics Online 1: 47–50. PMC2658868 19325852

[60] Librado P, Rozas J (2009) DnaSP v5: a software for comprehensive analysis of DNA polymorphism data. Bioinformatics 25: 1451–1452 doi:10.1093/bioinformatics/btp187. 19346325 10.1093/bioinformatics/btp187

[61] Tajima F (1989) Statistical Method for Testing the Neutral Mutation Hypothesis by DNA Polymorphism. Genetics 123: 585–595. 2513255 10.1093/genetics/123.3.585 PMC1203831

[62] Dereeper A, Guignon V, Blanc G, Audic S, Buffet S, et al (2008) Phylogeny.fr: robust phylogenetic analysis for the non-specialist. Nucleic Acids Res 36: W465–W469 doi:10.1093/nar/gkn180. 18424797 10.1093/nar/gkn180 PMC2447785

[63] Edgar RC (2004) MUSCLE: multiple sequence alignment with high accuracy and high throughput. Nucleic Acids Res 32: 1792–1797 doi:10.1093/nar/gkh340. 15034147 10.1093/nar/gkh340 PMC390337

[64] Guindon S, Gascuel O (2003) A Simple, Fast, and Accurate Algorithm to Estimate Large Phylogenies by Maximum Likelihood. Syst Biol 52: 696–704 doi:10.1080/10635150390235520. 14530136 10.1080/10635150390235520

[65] Chevenet F, Brun C, Bañuls A-L, Jacq B, Christen R (2006) TreeDyn: towards dynamic graphics and annotations for analyses of trees. BMC Bioinformatics 7: 439–448 doi:10.1186/1471-2105-7-439. 17032440 10.1186/1471-2105-7-439 PMC1615880

[66] Anisimova M, Gascuel O (2006) Approximate Likelihood-Ratio Test for Branches: A Fast, Accurate, and Powerful Alternative. Systematic Biology 55: 539–552 doi:10.1080/10635150600755453. 16785212 10.1080/10635150600755453

[67] Goloboff PA, Farris JS, Nixon KC (2008) TNT, a free program for phylogenetic analysis. Cladistics 24: 774–786 doi:10.1111/j.1096–0031.2008.00217.x.

[68] Gascuel O (1998) BIONJ: An Improved Version of the NJ Algorithm Based on a Simple Model of Sequence Data. Mol Biol Evol 14: 685–695. 10.1093/oxfordjournals.molbev.a025808 9254330

[69] Felsenstein J (1989) PHYLIP - Phylogeny Inference Package (Version 3.2). Cladistics 5: 164–166.

[70] Schuelke M (2000) An economic method for the fluorescent labeling of PCR fragments. Nat Biotechnol 18: 233–234. 10657137 10.1038/72708

[71] Lischer H (2009) PGDSpider v1.0.1.4. University of Bern, Bern, Switzerland.

[72] Szpiech ZA, Jakobsson M, Rosenberg NA (2008) ADZE: a rarefaction approach for counting alleles private to combinations of populations. Bioinformatics 24: 2498–2504 doi:10.1093/bioinformatics/btn478. 18779233 10.1093/bioinformatics/btn478 PMC2732282

[73] Goudet J (2001) FSTAT, a program to estimate and test gene diversities and fixation indices (version 2.9.3). Available at http://www2.unil.ch/popgen/softwares/fstat.htm.

[74] Rice WR (1989) Analyzing tables of statistical tests. Evolution 43: 223–225. 28568501 10.1111/j.1558-5646.1989.tb04220.x

[75] Raymond M, Rousset F (1995) GENEPOP (Version 1.2): Population Genetics Software for Exact Tests and Ecumenicism. J Hered 86: 248–249.

[76] Weir BS, Cockerham CC (1984) Estimating F-statistics for the analysis of population structure. Evolution 38: 1358–1370. 28563791 10.1111/j.1558-5646.1984.tb05657.x

[77] Evanno G, Regnaut S, Goudet J (2005) Detecting the number of clusters of individuals using the software structure: a simulation study. Mol Ecol 14: 2611–2620 doi:10.1111/j.1365–294X.2005.02553.x. 15969739 10.1111/j.1365-294X.2005.02553.x

