# Supplementary figures and images for: A European Melting Pot of Harbour Porpoise in the French Atlantic Coasts Inferred from Mitochondrial and Nuclear Data
Source: PLoS One. 2012 Sep 12;7(9):e44425. doi: 10.1371/journal.pone.0044425 (PMC3440431; doi:10.1371/journal.pone.0044425)

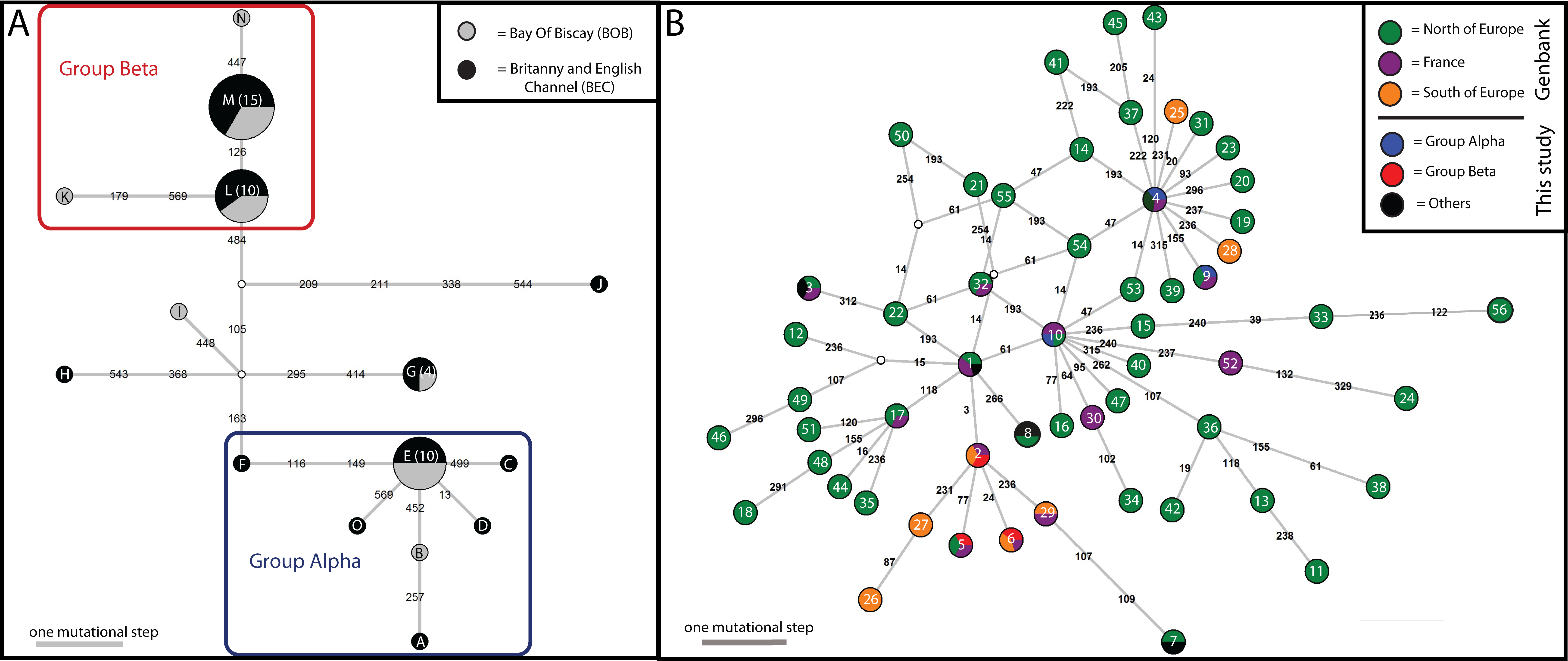

Supplement: Figure S1 — Mitochondrial haplotype networks. A. Haplotype network depicting the relationships between the 15 harbour porpoises mtDNA control region haplotypes determined in this study. B. Haplotype network of the 56 truncated haplotypes of mtDNA control region of harbour porpoise determined in this study and in previous ones. (TIF) [file pone.0044425.s001.tif]

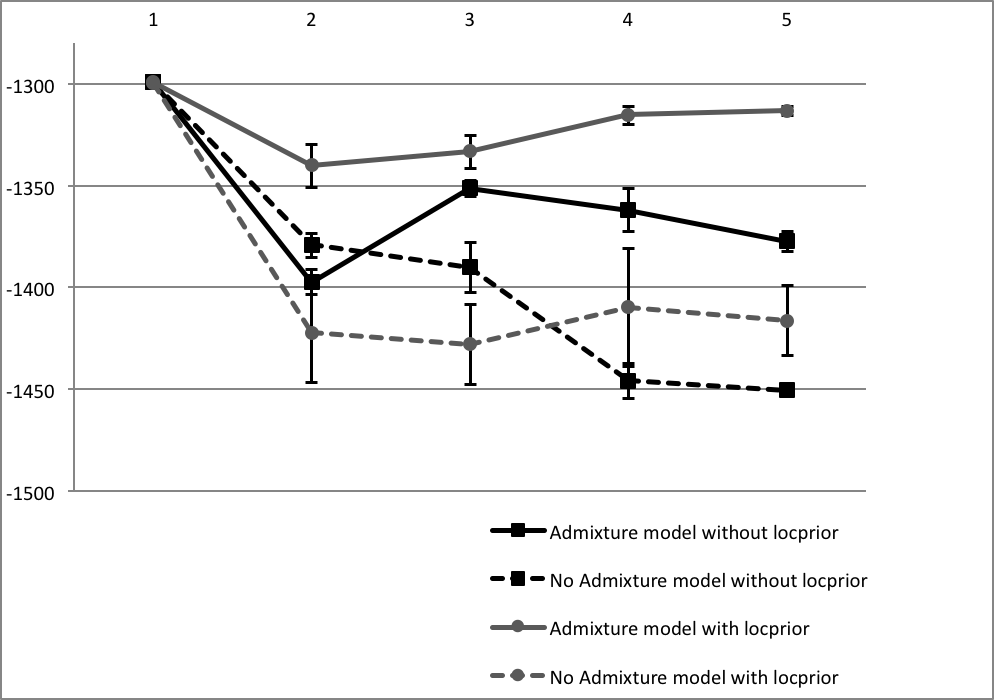


K

Ln Pr(XK)

Supplement: Figure S2 — Mean probabilities [LnPr(X|K)] of the data as a function of the fixed number of clusters (K). (DOC) [file pone.0044425.s002.doc]
