# Supplementary material for: A European Melting Pot of Harbour Porpoise in the French Atlantic Coasts Inferred from Mitochondrial and Nuclear Data
Source: PLoS One. 2012 Sep 12;7(9):e44425. doi: 10.1371/journal.pone.0044425 (PMC3440431; doi:10.1371/journal.pone.0044425)
Supplement: Table S1 — List of the 52 harbour porpoises sampled. (DOCX) [file pone.0044425.s003.docx]

**Table S1: List of the 52 harbour porpoises sampled.**

The geographic area, the days of sampling and the gender are given for each individual. The haplotypes are those determined in this study. Territorial and administrative division of France (from North to South): D62, “Pas de Calais”; D76, “Seine Maritime”; D22 “Côtes d’Armor”; D29, “Finistère”; D56, “Morbihan”; D85 “Vendée”; D17, “Charente-Maritime”; D33, “Gironde”; D40, “Landes”. ND: Non-determined.

| Geographic area of sampling | Individual | Stranding Date | Gender | Territorial and administrative division of France | Haplotype |
| --- | --- | --- | --- | --- | --- |
| **Brittany and English Channel** | ppbec1 | 24/01/2000 | male | D 29 | FrG |
|  | ppbec2 | 12/09/2004 | female | D 29 | FrM |
|  | ppbec3 | 11/12/2004 | female | D 29 | FrG |
|  | ppbec4 | 08/01/2005 | female | D 56 | FrO |
|  | ppbec5 | 04/03/2005 | female | D 76 | FrE |
|  | ppbec6 | 14/03/2005 | female | D 62 | FrD |
|  | ppbec7 | 01/10/2005 | male | D 29 | FrM |
|  | ppbec8 | 21/03/2006 | male | D 22 | FrJ |
|  | ppbec9 | 08/05/2006 | male | D 29 | FrE |
|  | ppbec10 | 05/10/2006 | male | D 29 | FrM |
|  | ppbec11 | 11/10/2006 | male | D 29 | FrM |
|  | ppbec12 | 19/01/2007 | female | D 56 | FrL |
|  | ppbec13 | 25/02/2007 | female | D 29 | FrG |
|  | ppbec14 | 22/09/2007 | male | D 29 | FrH |
|  | ppbec15 | 26/09/2007 | male | D 29 | FrM |
|  | ppbec16 | 03/11/2007 | female | D 29 | FrA |
|  | ppbec17 | 07/12/2007 | male | D 29 | FrM |
|  | ppbec18 | 07/02/2008 | female | D 56 | FrM |
|  | ppbec19 | 30/04/2008 | male | D 29 | FrL |
|  | ppbec20 | 24/07/2008 | male | D 22 | FrM |
|  | ppbec21 | 16/08/2008 | female | D 29 | FrL |
|  | ppbec22 | 07/09/2008 | male | D 29 | FrL |
|  | ppbec23 | 12/09/2008 | female | D 29 | FrL |
|  | ppbec24 | 15/09/2008 | female | D 29 | FrE |
|  | ppbec25 | 06/10/2008 | male | D 29 | FrE |
|  | ppbec26 | 25/12/2008 | male | D 29 | FrE |
|  | ppbec27 | 04/06/2009 | female | D 29 | FrM |
|  | ppbec28 | 10/09/2009 | female | D 29 | FrL |
|  | ppbec29 | 15/09/2009 | female | D 29 | FrC |
|  | ppbec30 | 19/03/2010 | female | D 29 | FrF |
|  | ppbec31 | 08/04/2010 | male | D 29 | FrM |
| **Bay of Biscay** | ppbob1 | 16/01/2000 | female | D 33 | FrI |
|  | ppbob2 | 01/03/2000 | female | D 85 | FrL |
|  | ppbob3 | 01/03/2000 | female | D 33 | FrG |
|  | ppbob4 | 13/03/2000 | male | D 33 | FrE |
|  | ppbob5 | 13/03/2000 | male | D 33 | FrL |
|  | ppbob6 | 25/02/2004 | female | D 33 | FrK |
|  | ppbob7 | 03/03/2004 | male | D 33 | FrM |
|  | ppbob8 | 05/03/2004 | male | D 33 | FrE |
|  | ppbob9 | 21/04/2004 | male | D 33 | FrB |
|  | ppbob10 | 06/05/2004 | female | D 40 | FrE |
|  | ppbob11 | 29/11/2004 | female | D 33 | FrM |
|  | ppbob12 | 08/01/2006 | male | D 33 | FrL |
|  | ppbob13 | 10/01/2006 | male | D 33 | FrE |
|  | ppbob14 | 13/01/2006 | male | D 33 | FrE |
|  | ppbob15 | 14/01/2006 | male | D 33 | FrM |
|  | ppbob16 | 18/01/2006 | male | D 33 | FrN |
|  | ppbob17 | 19/01/2006 | male | D 17 | FrL |
|  | ppbob18 | 02/02/2006 | female | D 33 | FrM |
|  | ppbob19 | 22/02/2006 | male | D 40 | FrM |
|  | ppbob20 | 09/01/2006 | male | D 33 | ND |
|  | ppbob21 | 16/06/2006 | male | D 17 | ND |
