# Supplementary material for: A European Melting Pot of Harbour Porpoise in the French Atlantic Coasts Inferred from Mitochondrial and Nuclear Data
Source: PLoS One. 2012 Sep 12;7(9):e44425. doi: 10.1371/journal.pone.0044425 (PMC3440431; doi:10.1371/journal.pone.0044425)
Supplement: Table S2 — Variable sites in the 15 mtDNA control region haplotypes defined in this study. (DOCX) [file pone.0044425.s004.docx]

**Table S2: Variable sites in the fifteen-mtDNA control region haplotypes defined in this study.** Only the variable nucleotide positions are represented. Sequences of the haplotype FrA is on the top row, and for the other haplotypes, identities with FrA are indicated by a dot, gaps (only for nt 105 and 154) by a dash. **N**: number of individuals sharing the haplotype. The numbering was established in function of the sequence determined in this study; position 1 is equivalent to position 15375 of the complete harbour porpoise mitochondrial genome sequence (Genbank accession AJ554063.1). Group alpha and beta have been identified during this study.

|  |  | Position | | | | | | | | | | | | | | | | | | | | | | | |  | |  |
| --- | --- | --- | --- | --- | --- | --- | --- | --- | --- | --- | --- | --- | --- | --- | --- | --- | --- | --- | --- | --- | --- | --- | --- | --- | --- | --- | --- | --- |
|  |  | **13** | **104** | **105** | **116** | **126** | **149** | **154** | **163** | **179** | **209** | **211** | **257** | **295** | **338** | **368** | **414** | **447** | **448** | **452** | **484** | **499** | **543** | **544** | **569** | | **N** |  |
| Haplotype names | **FrA** | C | A | G | A | G | C | A | C | G | G | T | C | C | C | C | A | T | A | A | C | A | C | T | C | | 1 | Group alpha |
|  | **FrB** | . | . | . | . | . | . | . | . | . | . | . | T | . | . | . | . | . | . | . | . | . | . | . | . | | 1 |  |
|  | **FrC** | . | . | . | . | . | . | . | . | . | . | . | T | . | . | . | . | . | . | G | . | . | . | . | . | | 1 |  |
|  | **FrD** | T | . | . | . | . | . | . | . | . | . | . | T | . | . | . | . | . | . | G | . | T | . | . | . | | 1 |  |
|  | **FrO** | . | . | . | . | . | . | . | . | . | . | . | T | . | . | . | . | . | . | G | . | . | . | . | T | | 1 |  |
|  | **FrE** | . | . | . | . | . | . | . | . | . | . | . | T | . | . | . | . | . | . | G | . | . | . | . | . | | 10 |  |
|  | **FrF** | . | . | . | G | . | T | . | . | . | . | . | T | . | . | . | . | . | . | G | . | . | . | . | . | | 1 |  |
|  | **FrG** | . | . | . | G | . | T | . | T | . | . | . | T | T | . | . | G | . | . | G | . | . | . | . | . | | 4 |  |
|  | **FrH** | . | . | . | G | . | T | _ | T | . | . | . | T | . | . | T | . | . | . | G | . | . | T | . | . | | 1 |  |
|  | **FrI** | . | **_** | . | G | . | T | . | T | . | . | . | T | . | . | . | . | . | G | G | . | . | . | . | . | | 1 |  |
|  | **FrJ** | . | . | A | G | . | T | . | T | . | A | C | T | . | T | . | . | . | . | G | . | . | . | C | . | | 1 |  |
|  | **FrK** | . | . | A | G | . | T | . | T | A | . | . | T | . | . | . | . | . | . | G | T | . | . | . | T | | 1 | Group beta |
|  | **FrL** | . | . | A | G | . | T | . | T | . | . | . | T | . | . | . | . | . | . | G | T | . | . | . | . | | 10 |  |
|  | **FrM** | . | . | A | G | A | T | . | T | . | . | . | T | . | . | . | . | . | . | G | T | . | . | . | . | | 15 |  |
|  | **FrN** | . | . | A | G | A | T | . | T | . | . | . | T | . | . | . | . | C | . | G | T | . | . | . | . | | 1 |  |
