# Supplementary material for: A European Melting Pot of Harbour Porpoise in the French Atlantic Coasts Inferred from Mitochondrial and Nuclear Data
Source: PLoS One. 2012 Sep 12;7(9):e44425. doi: 10.1371/journal.pone.0044425 (PMC3440431; doi:10.1371/journal.pone.0044425)
Supplement: Table S3 — List of the mtDNA control regions haplotypes previously determined by other authors on harbour porpoises and used in this study. (DOCX) [file pone.0044425.s005.docx]

**Table S3 : List of the mtDNA control regions haplotypes previously determined by other authors on harbour porpoises and used in this study.**

Sequences of the haplotypes were all published on Genbank, and authors mentioned the geographical origins of the animals.

| **Authors** | **Number of Haplotypes** | **Haplotype names** | **Genbank Accession**  **(from -> to)** | **Geographical origin** |
| --- | --- | --- | --- | --- |
| Tolley & Rosel, 2006 [39] | 18 | N1, S1, S2, S3, S4, S5, S6, S7, S8, S9, S10, S11, S12, S13, S14, S15, S16, S17 | AY262369.1-> AY262386.1 | Norway, North Sea, France, Portugal, Africa |
| Tolley et al. 2001 [36] | 10 | N3, N4, N5, N20, N21, N23, N24, IC1, IC5, IC14 | AF311924.1 -> AF311933.1 | Norway, Iceland |
| Tolley et al. , 2001 [36] | 14 | IC2, IC10, IC11, IC13, IC16, IC18, IC20, IC22, IC23, IC27, IC28, IC29, IC30, IC31, | GQ338845.1 -> GQ338858.1 | Iceland |
| Tolley & Rosel, 2006 [39] | 17 | N2, N6, N7, N8, N9, N10, N11, N13, N14, N15, N16, N17, N19, N22, N25, N26, N27 | GQ338859.1 -> GQ338875.1 | Norway |
| Viaud-Martinez et al., 2007 [40] | 13 | Via19, Via20, VIa21, Via22, Via 23, Via24, Via 25, Via26, Via 27, Via 28, Via 29, Via 30, Via 31 | EF063664.1 -> EF063676.1 | France, Gibraltar |
| Tiedemann et al., 1996 [32] | 9 | Pho1, Pho2, Pho3, Pho4, Pho5, Pho6, Pho7, Pho8, Pho9 | Y13872.1 -> Y13880.1 | North sea, baltic Sea |
| Walton, 1997 [31] | 1 | UK1 | X91613.1 | United Kingdom |
