# Supplementary material for: A European Melting Pot of Harbour Porpoise in the French Atlantic Coasts Inferred from Mitochondrial and Nuclear Data
Source: PLoS One. 2012 Sep 12;7(9):e44425. doi: 10.1371/journal.pone.0044425 (PMC3440431; doi:10.1371/journal.pone.0044425)
Supplement: Table S4 — Haplotype data sets analyzed. (DOCX) [file pone.0044425.s006.docx]

**Table S4. Haplotype data sets analyzed**

FrA to FrN are the 15 haplotypes of mtDNA Control Region determined in this study. They were truncated from 581 bp to a 334 bp fragment, common with the sequences determined previously by others and published in Genbank. Names of the corresponding haplotypes and references for the published sequences are given.

| **Haplotypes (this study)** | **Group** | **Corresponding truncated haplotype** | **Corresponding published sequences (geographical origin of the sample)** | **Authors** |
| --- | --- | --- | --- | --- |
| FrA | Group α | FrAt | N3 (Norway); VIA 19 (France) | (Tolley & P E Rosel, 2006) [39]  (Viaud-Martinez et al., 2007) [40] |
| FrB, FrC, FrD, FrE, FrO |  | FrEt | PH07, PH01 (North Sea); S11 (France); N1 (North Sea, France, Norway) ; VIA 29 (France); UK1 (United Kingdom) | (Tiedemann et al., 1996) [32]  (Tolley & P E Rosel, 2006) [39]  (Viaud-Martinez et al., 2007) [40]  (M. J. Walton, 1997) [31] |
| FrF |  | FrFt | S12 (France); VIA 20 (France); S1 (France, North Sea) | (Tolley & P E Rosel, 2006) [39]  (Viaud-Martinez et al., 2007) [40] |
| FrG | unassigned | FrGt | S4 (France, North Sea); VIA 22 (France) | (Tolley & P E Rosel, 2006) [39]  (Viaud-Martinez et al., 2007) [40] |
| FrH |  | FrHt | IC5 (Iceland) | (Tolley et al. 2001) [36] |
| FrI |  | FrIt | N16 (Norway); S7 (France); N4 (Norway); VIA 25 (France); | (Tolley & P E Rosel, 2006) [39]  (Viaud-Martinez et al., 2007) [40] |
| FrJ |  | FrJt | N15 (Norway) | (Tolley & P E Rosel, 2006) [39] |
| FrK | Group β | FrKt | S5 (North Sea); VIA 26 (France) | (Tolley & P E Rosel, 2006) [39]  (Viaud-Martinez et al., 2007) [40] |
| FrL |  | FrLt | S8 (Portugal, France); VIA 27 (France) | (Tolley & P E Rosel, 2006) [39]  (Viaud-Martinez et al., 2007) [40] |
| FrM, FrN |  | FrMt | S9 (France) ; S6 (Portugal, France); VIA 28 (France) | (Tolley & P E Rosel, 2006) [39]  (Viaud-Martinez et al., 2007) [40] |
