# Supplementary material for: A European Melting Pot of Harbour Porpoise in the French Atlantic Coasts Inferred from Mitochondrial and Nuclear Data
Source: PLoS One. 2012 Sep 12;7(9):e44425. doi: 10.1371/journal.pone.0044425 (PMC3440431; doi:10.1371/journal.pone.0044425)
Supplement: Table S5 — Summary statistics for the 7 microsatellite loci analyzed and for each group of samples. (DOCX) [file pone.0044425.s007.docx]

**Table S5:** **Summary statistics for the 7 microsatellite loci analyzed and for each group of samples**

Data are expressed for each locus and as the average of all loci for (a) all samples, (b) arbitrary geographic groups and (c) genetic groups α and β defined during mtDNA control region sequence analysis.

N = sample size, nA = number of alleles, range = range of allele sizes in bp, *He* = non biased expected heterozygosity, *Ho* = observed heterozygosity, A = allelic richness (estimated for a sample size of 14 individuals), *F_is_* _=_ value *F_is_* calculated after Weir and Cockerham. Asterisks mark significant departure from HWE after the Bonferroni correction (* : p < 0.05)

|  | **N** | **nA** | **range** | ***He*** | ***Ho*** | **A** | ***Fis*** |
| --- | --- | --- | --- | --- | --- | --- | --- |
| **(a) all  samples** | | | | | | | |
| PPHO110 | 46 | 9 | 124 - 146 | 0.795 | 0.891 | 6.35 | -0.122 |
| PPHO130 | 45 | 15 | 113 - 143 | 0.834 | 0.844 | 10.56 | -0.013 |
| PPHO137 | 45 | 15 | 159 - 189 | 0.885 | 0.756 | 10.24 | 0.148 |
| PPHO102 | 46 | 10 | 176 - 198 | 0.670 | 0.457 | 7.48 | 0.321* |
| PPHO142 | 46 | 15 | 174 - 208 | 0.789 | 0.826 | 9.60 | -0.048 |
| PPHO104 | 46 | 13 | 193 - 233 | 0.860 | 0.891 | 9.30 | -0.037 |
| PPHO131 | 46 | 7 | 110 - 130 | 0.809 | 0.913 | 5.81 | -0.130 |
| all loci | 46 | 12.0 |  | 0.806 | 0.797 | 8.48 | 0.011 |
|  |  |  |  |  |  |  |  |
| **(b) Geographic arbitrary groups** | | | | | | | |
| **Group BOB** |  |  |  |  |  |  |  |
| PPHO110 | 20 | 9 | 124 - 146 | 0.822 | 0.900 | 7.71 | -0.098 |
| PPHO130 | 19 | 12 | 115 - 143 | 0.859 | 0.842 | 10.53 | 0.020 |
| PPHO137 | 19 | 13 | 159 - 189 | 0.892 | 0.895 | 11.55 | -0.003 |
| PPHO102 | 20 | 8 | 178 - 196 | 0.654 | 0.500 | 7.34 | 0.240 |
| PPHO142 | 20 | 12 | 174 - 206 | 0.836 | 0.850 | 10.03 | -0.017 |
| PPHO104 | 20 | 11 | 201 - 233 | 0.895 | 0.950 | 9.91 | -0.063 |
| PPHO131 | 20 | 7 | 110 - 130 | 0.818 | 0.900 | 6.38 | -0.103 |
| all loci | 20 | 10.3 |  | 0.825 | 0.834 | 9.06 | -0.011 |
| **Group BEC** |  |  |  |  |  |  |  |
| PPHO110 | 26 | 6 | 132 -144 | 0.783 | 0.885 | 5.52 | -0.133 |
| PPHO130 | 26 | 13 | 113 - 141 | 0.808 | 0.846 | 10.03 | -0.049 |
| PPHO137 | 26 | 10 | 159 - 183 | 0.825 | 0.654 | 8.39 | 0.211 |
| PPHO102 | 26 | 9 | 176 - 198 | 0.686 | 0.423 | 7.59 | 0.388* |
| PPHO142 | 26 | 13 | 174 - 208 | 0.753 | 0.808 | 9.95 | -0.074 |
| PPHO104 | 26 | 11 | 193 - 231 | 0.826 | 0.846 | 8.97 | -0.025 |
| PPHO131 | 26 | 6 | 116 - 130 | 0.814 | 0.923 | 5.54 | -0.137 |
| all loci | 26 | 9.7 |  | 0.785 | 0.769 | 8.00 | 0.020 |
|  |  |  |  |  |  |  |  |
| **(c) Genetic groups α and β defined with mtDNA control region sequence analysis** | | | | | | | |
| **Group α** |  |  |  |  |  |  |  |
| PPHO110 | 14 | 7 | 130 - 144 | 0.833 | 0.857 | 7.00 | -0.030 |
| PPHO130 | 14 | 11 | 113 - 141 | 0.862 | 0.929 | 11.00 | -0.080 |
| PPHO137 | 14 | 9 | 167 - 185 | 0.844 | 0.643 | 9.00 | 0.245 |
| PPHO102 | 14 | 7 | 176 - 198 | 0.709 | 0.429 | 7.00 | 0.405 |
| PPHO142 | 14 | 9 | 174 - 204 | 0.786 | 0.786 | 9.00 | 0.000 |
| PPHO104 | 14 | 9 | 201- 225 | 0.857 | 0.714 | 9.00 | 0.172 |
| PPHO131 | 14 | 6 | 110 - 126 | 0.815 | 0.929 | 6.00 | -0.146 |
| all loci | 14 | 8.3 |  | 0.815 | 0.755 | 8.29 | 0.076 |
| **Group β** |  |  |  |  |  |  |  |
| PPHO110 | 23 | 8 | 124 - 146 | 0.811 | 0.957 | 6.77 | -0.185 |
| PPHO130 | 22 | 12 | 115 - 141 | 0.851 | 0.818 | 10.37 | 0.039 |
| PPHO137 | 23 | 15 | 159 - 189 | 0.899 | 0.870 | 11.65 | 0.033 |
| PPHO102 | 23 | 9 | 176 - 196 | 0.703 | 0.522 | 7.59 | 0.263 |
| PPHO142 | 23 | 13 | 174 - 206 | 0.821 | 0.870 | 10.52 | -0.060 |
| PPHO104 | 23 | 12 | 193 - 233 | 0.876 | 0.957 | 9.83 | -0.094 |
| PPHO131 | 23 | 6 | 116 - 130 | 0.814 | 0.913 | 5.60 | -0.125 |
| all loci | 23 | 10.7 |  | 0.825 | 0.844 | 8.90 | -0.023 |
