# Supplementary material for: A European Melting Pot of Harbour Porpoise in the French Atlantic Coasts Inferred from Mitochondrial and Nuclear Data
Source: PLoS One. 2012 Sep 12;7(9):e44425. doi: 10.1371/journal.pone.0044425 (PMC3440431; doi:10.1371/journal.pone.0044425)
Supplement: Table S6 — Correspondances between truncated haplotypes and their coding on ML phylogenetic tree and on haplotype networks. (DOCX) [file pone.0044425.s008.docx]

**Table S6 : Correspondances between truncated haplotypes and their coding on ML phylogenetic tree and on haplotype networks**

All identical haplotypes after truncation are grouped on a same lane

| **Haplotype(s)** | **Number of haplotypes** | **Leaf name on the phylogenetic tree** | **Name of the haplotype network** |
| --- | --- | --- | --- |
| FrIt N16 S7 N4 VIA25 | 5 | N4 | 1 |
| FrLt S8 VIA27 | 3 | S8 | 2 |
| FrGt S4 VIA22 | 3 | S4 | 3 |
| FrEt FrBt FrOt FrDt FrCt S11 N1 VIA29 UK1 PHO7 PHO1 | 11 | N1 | 4 |
| FrKt S5 VIA26 | 3 | S5 | 5 |
| FrNt FrMt S9 S6 VIA28 | 5 | S6 | 6 |
| FrJt N15 | 2 | N15 | 7 |
| FrHt IC5 | 2 | IC5 | 8 |
| FrAt N3 VIA19 | 3 | N3 | 9 |
| FrFt S12 S1 VIA20 | 4 | S1 | 10 |
| N27 | 1 | N27 | 11 |
| N9 | 1 | N9 | 12 |
| N2 | 1 | N2 | 13 |
| N26 PHO2 | 2 | N26 | 14 |
| N25 | 1 | N25 | 15 |
| N22 N11 | 2 | N22 | 16 |
| N19 N20 VIA24 | 3 | N19 | 17 |
| N17 | 1 | N17 | 18 |
| N14 | 1 | N14 | 19 |
| N13 | 1 | N13 | 20 |
| N10 | 1 | N10 | 21 |
| N8 | 1 | N8 | 22 |
| N7 | 1 | N7 | 23 |
| N6 | 1 | N6 | 24 |
| S17 | 1 | S17 | 25 |
| S16 VIA30 | 2 | S16 | 26 |
| S15 | 1 | S15 | 27 |
| S14 | 1 | S14 | 28 |
| S13 VIA31 | 2 | S13 | 29 |
| S10 VIA21 | 2 | S10 | 30 |
| S3 | 1 | S3 | 31 |
| S2 PHO6 PHO4 | 3 | S2 | 32 |
| IC14 | 1 | IC14 | 33 |
| N24 | 1 | N24 | 34 |
| N23 | 1 | N23 | 35 |
| N21 | 1 | N21 | 36 |
| N5 | 1 | N5 | 37 |
| IC31 | 1 | IC31 | 38 |
| IC30 | 1 | IC30 | 39 |
| IC29 | 1 | IC29 | 40 |
| IC28 | 1 | IC28 | 41 |
| IC27 | 1 | IC27 | 42 |
| IC23 | 1 | IC23 | 43 |
| IC22 | 1 | IC22 | 44 |
| IC20 | 1 | IC20 | 45 |
| IC18 | 1 | IC18 | 46 |
| IC16 | 1 | IC16 | 47 |
| IC13 | 1 | IC13 | 48 |
| IC11 | 1 | IC11 | 49 |
| IC10 | 1 | IC10 | 50 |
| IC2 | 1 | IC2 | 51 |
| VIA23 | 1 | VIA23 | 52 |
| PHO9 | 1 | PHO9 | 53 |
| PHO8 PHO3 | 2 | PH03 | 54 |
| PHO5 | 1 | PHO5 | 55 |
| IC1 | 1 | IC1 | 56 |
| ViA1 | 1 | Via 1 | absent of network |
