# Supplementary material for: A European Melting Pot of Harbour Porpoise in the French Atlantic Coasts Inferred from Mitochondrial and Nuclear Data
Source: PLoS One. 2012 Sep 12;7(9):e44425. doi: 10.1371/journal.pone.0044425 (PMC3440431; doi:10.1371/journal.pone.0044425)
Supplement: Table S7 — Primer sequences for the seven microsatellites loci analyzed in this study. (DOCX) [file pone.0044425.s009.docx]

**Table S7: Primer sequences for the seven microsatellites loci analyzed in this study.** Locus names are from (P. E. Rosel et al., 1999). Tails corresponding to universal primers are underlined. Universal primers sequences were GTCGTAGTCGACGACCGTTA (linked to FAM) and GACTTCGGATAGCTAGTCGT (linked to Hex). Forward (f) and reverse (r) are given by reference to the Genbank sequences.

| **Locus name/**  **Genbank Accession** | **Primer names** | **Primer sequences (5’-3’)** |
| --- | --- | --- |
| PPHO110  /AF151785 | Pp HO110EAf | CTACACAAACCTATAAATGAGATAAA |
|  | Pp HO110EAr | TAGTCGACGACCGTTAGACCTTATTCCGTCTTCACC |
| PPHO130  /AF151786 | Pp HO130EAf | CTACACACACCTCTATTCAAGCCC |
|  | Pp HO130EAr | TCGGATAGCTAGTCGTAGAATGTCCAACACAGGAGCAG |
| PPHO137  /AF151787 | PpHO137EAf | AGTGTGCAGAGAAATGGCCTCA |
|  | PpHO137EAr | TCGGATAGCTAGTCGTAGCTTGGAGTTTGGCTCCCT |
| PPHO102/AF151788 | Pp HO102EAf | CCTATCAACACCCTGGAGTTATGC |
|  | Pp HO102EAr | TCGGATAGCTAGTCGTAAAGGAGAACAGGAGGAGGAGG |
| PPHO142/AF151789 | Pp HO142EAf | AAATAACACACACGGCCGCA |
|  | Pp HO142EAr | TAGTCGACGACCGTTAAACCGTTCCCAACTTGGTTTGC |
| PPHO104/AF151790 | Pp HO104EAf | TCGGATAGCTAGTCGTTGTGTGGCTGAGAAAGCACTGA |
|  | Pp HO104EAr | TTTATGGGCCTGCTGGGTACAA |
| PPHO131/AF151791 | Pp HO131EAf | TAGTCGACGACCGTTAGCCATCAATCAGGACAGCACCTA |
|  | Pp HO131EAr | GCGCTTGGAGAGATTTCGACGTA |
